# High Endogenous Expression of Chitinase 3-Like 1 and Excessive Epithelial Proliferation with Colonic Tumor Formation in MOLF/EiJ Mice

**DOI:** 10.1371/journal.pone.0139149

**Published:** 2015-10-06

**Authors:** Daren Low, Arianna K. DeGruttola, Alexander Poltrak, Atsushi Mizoguchi, Mari Mino-Kenudson, Emiko Mizoguchi

**Affiliations:** 1 Gastrointestinal Unit, Department of Medicine, Massachusetts General Hospital and Harvard Medical School, Boston, MA, United States of America; 2 Department of Molecular Biology, Petrozavodsk State University, 185910, Petrozavodsk, Republic of Karelia, Russia; 3 Graduate Program in Genetics, Sackler School of Biomedical Sciences, Tufts University, Boston, MA, United States of America; 4 Department of Immunology, Kurume University School of Medicine, Kurume, Fukuoka, Japan; 5 Department of Pathology, Massachusetts General Hospital and Harvard Medical School, Boston, MA, United States of America; 6 Center for the Study of Inflammatory Bowel Disease, Massachusetts General Hospital and Harvard Medical School, Boston, MA, United States of America; Indiana University School of Medicine, UNITED STATES

## Abstract

Colorectal cancer (CRC) development is mediated by uncontrolled survival and proliferation of tumor progenitor cells. Using animal models to identify and study host-derived factors that underlie this process can aid interventions in preventing tumor expansion and metastasis. In healthy steady states in humans and mice (e.g. C57BL/6 strain), colonic Chitinase 3-like 1 (CHI3L1) gene expression is undetectable. However, this expression can be induced during intestinal inflammation and tumorigenesis where CHI3L1 plays an important role in tissue restitution and cell proliferation. Here, we show that a wild-derived mouse strain MOLF/EiJ expresses high levels of colonic epithelial CHI3L1 at the steady state due to several nucleotide polymorphisms in the proximal promoter regions of the CHI3L1 gene. Interestingly, these mice spontaneously developed polypoid nodules in the colon with signs of immune cell infiltrations at steady state. The CHI3L1 positive colonic epithelial cells were highly proliferative and exhibited malignant transformation and expansion when exposed *in vivo* to azoxymethane, one of the well-known colonic carcinogens.

## Introduction

Colorectal cancer (CRC) is one of the most common cancers, accounting for approximately 600,000 deaths a year globally [**1**]. CRC can develop either via an adenoma-carcinoma sequence or an inflammation-dysplasia-carcinoma sequence. Although the precise mechanisms behind the two forms of CRCs may be different, the basic prerequisite for tumor expansion in both cases involves uncontrolled proliferation and survival of colonic epithelial cells (CECs).

Chitinase 3-like 1 (CHI3L1) is a pseudo-chitinase that is undetectable in the colon in healthy individuals, but its expression is induced during the course of intestinal inflammation and CRC development [**2,3**]. In the colon, CHI3L1 expression primarily serves to promote proliferation and survival of CECs and endothelial cells. Inhibition of CHI3L1 using pan-chitinase inhibitors, including cyclic dipeptide CI–4 [cyclo-(I-Arg-d-Pro)], allosamidine, and methylxanthine derivatives (e.g. caffeine), effectively retard the growth and viability of colon cancer cell line [**4,5**]. This cell survival and proliferative effects of CHI3L1 subsequently promote higher cancer cell migration/metastasis with an increased risk of malignancy. Transgenic (Tg) mice overexpressing CHI3L1 that received melanoma cells through tail vein injection developed extensive metastatic melanoma colonies in the lungs [**6**]. In addition to CRCs, up-regulation of CHI3L1 has been widely suggested to be a biomarker for a wide range of solid tumors including breast and lung cancers [**7**]. All of these clearly support the pro-tumorigenic effect of CHI3L1; once its expression is induced, it promotes cell survival and proliferation.

Genetic engineering of common laboratory mouse strains has been an instrumental model to study the mechanism of CRC pathogenesis. Currently, several mice lines, including the Adenomatous polyposis coli (APC) knockout (KO) mice and Tg mice containing the Kras^V12G^ point mutation are available. On a C57/Bl6 (B6) background Apc^min^ (Min, multiple intestinal neoplasia) mice, which have a point mutation in the murine homology of the APC gene are highly susceptible to the development of spontaneous intestinal adenoma [**8**]. Similarly, up to 80% of Kras^V12G^ Tg mice on a B6D2 background showed aberrant crypt foci and/or invasive adenocarcinomas [**9**]. In addition, murine colitis models that spontaneously develop intestinal inflammation, such as IL–10 KO and Gαi2 KO mice, also show increased incidences of colonic adenocarcinoma, allowing better understanding of the inflammation-associated carcinogenic changes in CECs [**10**]. However, the challenge now is to better define and dissect the exact differences between genes that induce tumor transformation, as opposed to genes that may not have the potential to induce tumor transformation but rather promote survival and proliferation of both normal and neoplastic CECs.

In addition to classical inbred mouse strains, wild-derived strain of mice may provide an invaluable model to study disease pathogenesis due to the significant evolutionary distance that results in a range of genetic polymorphisms. These wild-derived strains of mice, such as the MOLF/EiJ (*Mus musculus molossinus*: abbreviated as MOLF), are usually descendants of captured wild mice and the genetic diversity of these wild-derived mice allows better phenotypic screenings and genetic mapping of disease causal genes. In this study, we identify that MOLF mice show high levels of colonic CHI3L1 expression under healthy steady state compared to B6 mice as the result of multiple polymorphisms at the CHI3L1 proximal promoter region. These MOLF mice develop spontaneous polypoid nodules in the colon under steady state that display excessive proliferation/survival of non-neoplastic CECs, recapitulating biological characteristics of endogenous CHI3L1. MOLF mice are more susceptible to azoxymethane (AOM)-induced carcinogenesis as compared to B6 mice.

## Materials and Methods

### Animals and ethic statement

The animal protocols (#2005N000254 and #2011N000017) have been reviewed and approved by the Massachusetts General Hospital Subcommittee on Research Animal Care (SRAC)-OLAW Assurance #A3596-01. These protocols as submitted and reviewed confirm to the USDA Animal Welfare Act, Partners Healthcare System Policy on Human Care and Use of Laboratory Animals, the Institute for Laboratory Animal Research Guide for the Care and Use of Laboratory Animals and applicable laws and regulations. Six weeks old MOLF and age-matched B6 mice were purchased from the Jackson Laboratory (Bar Harbor, ME, USA) and housed in the Massachusetts General Hospital specific pathogen free facility under a 12-hour day/night light cycle. Body weight and physical conditions were checked daily to monitor disease progression and animal wellbeing during the duration of experiments. All mice were non-fasting and had access to food and drinking water *ad libitum*. Mice were euthanized in accordance with SRAC guidelines by CO_2_ inhalation. No other analgesics/anesthetics were used in this study. No animals showed severe illness (e.g., more than 15% body weight loss, rectal prolapse) or die during the duration of experiments.

### Enzyme-linked immunosorbent assay (ELISA)

Mouse CHI3L1 levels in serum were detected using ELISA kit purchased from R&D systems (Minneapolis, MN, USA) and performed according to manufacturer’s instructions. Optical density was measured at 450 nm using Auto-Reader Model 680 (Bio-Rad, Hercules, CA, USA).

### Construction of MOLF- or B6- CHI3L1 promoter luciferase reporter plasmid

Six month old MOLF and C57Bl/6 colonic genomic DNA was extracted and the CHI3L1 proximal promoter was amplified using forward primer: 5’-TGAGTCACATCACCACAGTC and reverse primer 5’-GCCCTCCTTACAGGAACTG. The PCR product was individually cloned into the *Xho*I and *Hind*III site of the pGL3-basic luciferase reporter plasmid (Promega, Madison, WI, USA).

### Luciferase assay

2 X 10^4^ SW480 CECs were seeded on at 24 well plates. Empty pGL3 control plasmid (0.7 μg), MOLF-CHI3L1-promoter-pGL3 (0.7 μg), B6-CHI3L1-promoter-pGL3 (0.7 μg) were transfected to the seeded cells 24 hours later using Lipofectamine 2000 (Invitrogen), together with 0.1 μg of Renilla reporter plasmid. Cells were harvested 48 hours later and luciferase activities were measure using the Dual-Reporter Luciferase Assay System (Promega) according to manufacturer’s protocol.

### Tumor induction

Eight to ten weeks old B6 and MOLF mice (n = 7 from each group) were intraperitoneally injected with 12 mg/kg of azoxymethane (AOM; Sigma Aldrich) once a week for 6 weeks. After additional 8 weeks, mice were sacrificed and the colons were opened-up and macroscopic tumors were counted and the colon was examined to measure the number and size of macroscopic tumors. Colonic tissue was evaluated by 2 independent investigators (MMK and EM) in a blinded fashion. The tumor score represents the score 0 to 5 as follows: 0, no tumor; 1, low-grade dysplasia; 2, high-grade dysplasia; 3, intra-mucosal adenocarcinoma (carcinoma in situ), 4, invasive carcinoma; 5, distant metastasis [**11**].

### Immunohistochemical analysis

Colonic frozen sections (4 μm thick) were stained with anti-Ki67 (Abcam, Cambridge, MA, USA), rabbit anti-CHI3L1 (Affinity Bioreagent, Golden, CO), rat anti-F4/80 (Serotec, Raleigh, NC, USA), -CD4 (eBioscience, San Diego, CA, USA), -CD11b (BD Pharmingen, San Jose, CA, USA), or -cytokeratin 8, TROMA–1 (Developmental Studies Hybridoma Bank at the University of Iowa (Iowa City, IA) antibodies, as well as normal rabbit Ig using the avidin-biotin-complex system (Vector laboratories) as previously described [**12**].

### Confocal Microscopic analysis

Mouse colonic cryosections were fixed in acetone for 10 minutes and air-dried at RT, and blocked in 5% goat and horse serum for 30 minutes at RT and then co-stained with rabbit-anti-mouse/human CHI3L1 and rat-anti-CD4, -F4/80 or -CD11b antibodies at 4°C overnight. Sections were washed three times with PBS and stained with Alexa Fluor 647-conjugated goat anti-rabbit IgG (Invitrogen) and FITC-conjugated horse anti-mouse IgG secondary antibodies (Vector, Burlingame, CA) for 30 minutes at RT. The sections were washed three times with PBS, mounted in Vecta-shield (Vector) and analyzed under a Bio-Rad Radiance 2000 confocal microscope (Bio-Rad).

### Statistical Analysis

Statistics analyses were performed using Student’s T-test or non-parametric test (Mann-Whitney U test by utilizing GraphPad Prism 6 software, http://www.graphpad.com/scientific-software/prism/). Multiple comparison experiments were analyzed to determine statistical significance within individual groups. Values were presented in mean ± the standard error of the mean (SEM).

## Results

### MOLF mice express high level of colonic CHI3L1 at steady-state

**Fig 1:**
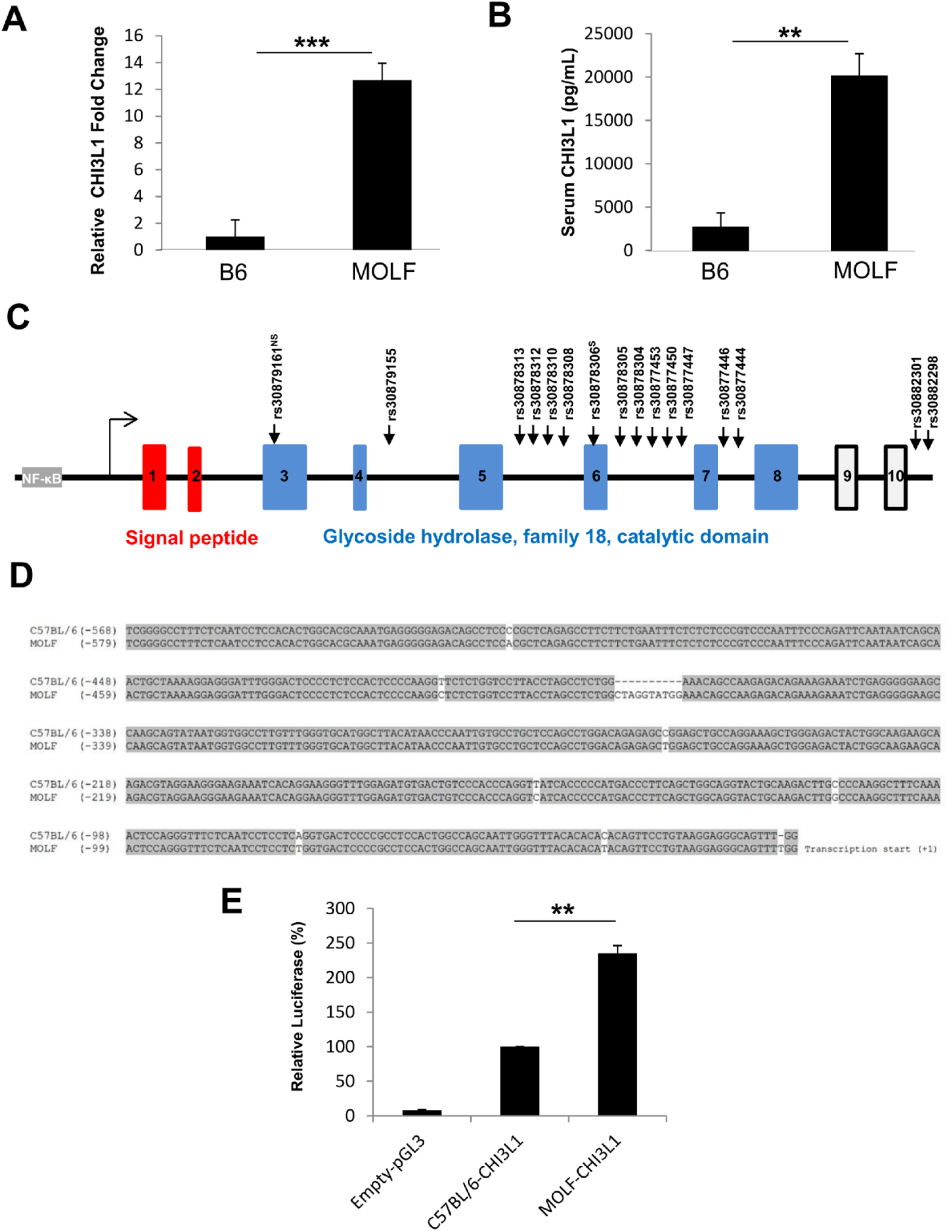
High endogenous colonic CHI3L1 expression state MOLF/EiJ mice colon at steady state. [**A**] Colonic CHI3L1 mRNA level in MOLF/Eij (MOLF) and C57BL/6 (B6) mice were measured using real time PCR (n = 5 in each group). [**B**] Serum CHI3L1 protein level in MOLF (n = 5) and B6 (n = 5) mice were quantified using ELISA. [**C**] Schema showing the genomic structure of the CHI3L1 locus. Arrows indicating single nucleotide polymorphisms between MOLF and B6 mice identified by in silico comparison using the Jackson mouse phenome database. [**D**] Proximal promoter from MOLF and B6 mice was PCR amplified, sequenced and aligned using Clustal W program. [**E**] SW480 cells were transfected with pGL3-basic vector containing MOLF- or B6-derived CHI3L1 proximal promoter and renilla-normalised luciferase assay was performed (n = 5 in each group). **P<0.01, ***P<0.001 between the two groups as indicated.

In the initial set of experiments, we unexpectedly found that CHI3L1 mRNA levels as measured by quantitative real time PCR were significantly increased in the colon of MOLF mice as compared to B6 mice at 6 months of age [**Fig 1A**]. This finding was consistent with the data from serum that shows higher levels of CHI3L1 protein in the MOLF mice as compared to age-matched B6 mice [**Fig 1B**].

**Fig 2:**
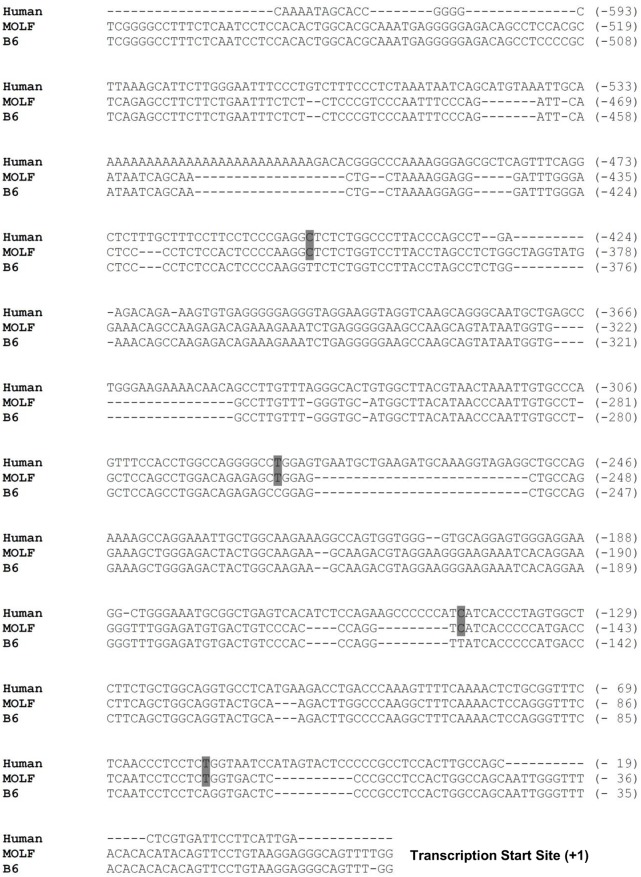
Alignment of Human, MOLF and B6 mice CHI3L1 proximal promoter. Approximately 600 bp upstream sequence of the CHI3L1 transcription start site from human, MOLF and B6 mice were aligned using Clustal W program.

To identify the genetic variations in both strains of mice on the CHI3L1 locus, CHI3L1 intragenic sequence was analyzed for single nucleotide polymorphisms (SNPs) using the Jackson Laboratory Mouse Phenome Database (http://phenome.jax.org). Sequence comparisons between B6 and MOLF mice show at least 16 known SNPs along the CHI3L1 intragenic locus (i.e. 14 in introns and 2 in exons) [**Fig 1C**]. Therefore, the proximal promoter regions of both strains of mice were PCR amplified and then sequenced. Indeed, there were 8 SNPs and an additional 10 base-pair nucleotide insertion in the CHI3L1 proximal promoter region of the MOLF mice [**Fig 1D**]. While comparing the CHI3L1 promoter sequence of these two mouse strains to the human counterpart, it is notable that these SNPs in the MOLF mice are more conserved to that of the human counterpart, rather than those of B6 mice [**Fig 2**].

To determine whether these promoter SNPs exert any effects on transcriptional activities, each MOLF and B6 proximal promoter region was individually cloned into the pGL3-luciferase reporter vector and transfected into SW480 human colonic CEC line. Subsequent luciferase assay after 48 hours revealed higher luciferase activity in the cell transfected with the MOLF-derived CHI3L1 promoter construct transfected cells, as compared to the B6-derived CHI3L1 promoter construct. These findings suggest that the genetic variation present in MOLF can enhance CHI3L1 promoter transcriptional activity [**Fig 1E**].

### MOLF mice spontaneously develop polypoid nodules in distal colon

**Fig 3:**
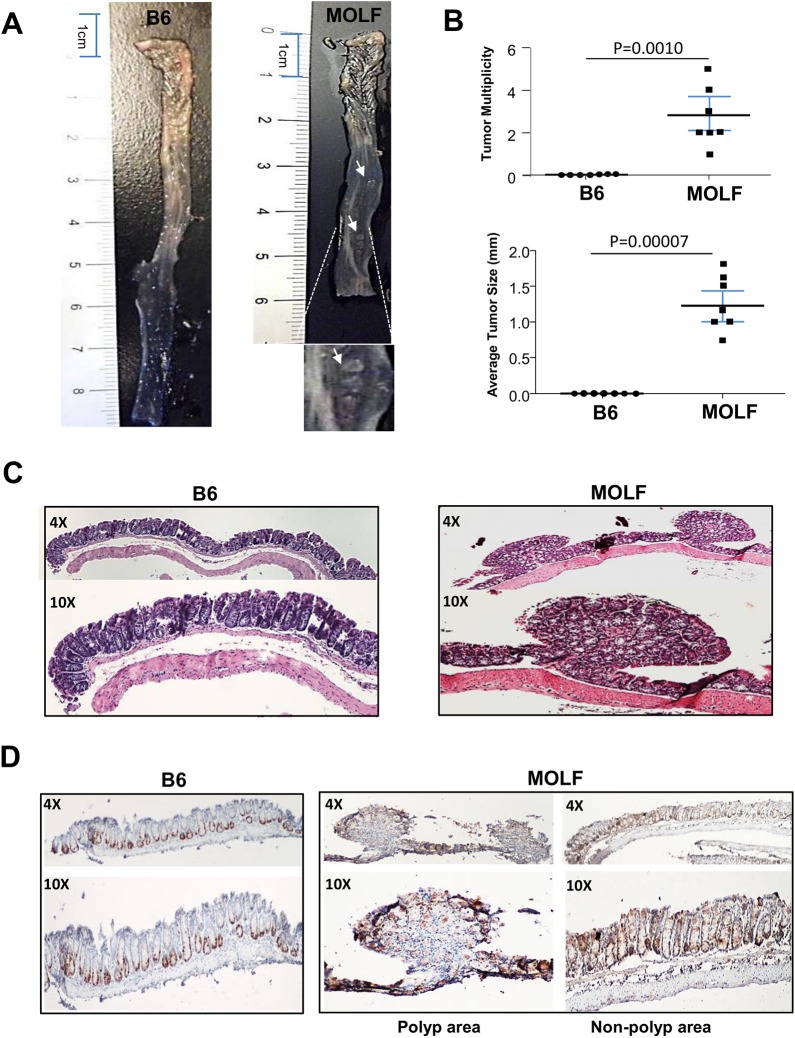
MOLF mice spontaneously developed polyp-like nodules in colon at steady state. [**A**] Six months old MOLF and B6 mice were sacrificed and the colons were opened up longitudinally. [**B**] Multiplicity and size of polyp-like nodules were determined under a dissection microscope (n = 7 in each group). [**C**] H&E sections of colons from B6 and MOLF are shown. [**D**] IHC staining of colonic sections from B6 and MOLF mice was performed using anti-Ki67 primary antibody.

To determine whether the presence of high CHI3L1 levels at steady-state renders any phenotypic aberrations in the MOLF mice, 6 months old MOLF and age-matched B6 control mice were euthanized and the whole colon was grossly examined. Surprisingly, protruding polypoid nodules were macroscopically detectable in almost all of the MOLF mice at steady-state predominately in the distal part of colon, but not in any of the age-matched B6 mice [**Fig 3A and 3B**]. These polypoid nodules were confirmed on the H&E sections from the colon of the MOLF mice, whereas no abnormality can be observed in the B6 mice. Although these protrusions appear to be highly conspicuous, pathological examination did not reveal any cytological abnormalities or dysplasia transformation. Conversely, an immunohistochemical analysis showed remarkably high Ki–67 labeling indices in the MOLF mice, consistent with hyper-proliferative rates in CECs [**Fig 3C and 3D**]. In the polypoid nodules, Ki–67 labeling was mainly seen in the tip of each nodule, suggesting a bottom-down expansion. In contrast, B6 mice displayed normal Ki–67 staining pattern, detected predominantly at the base of the individual intestinal crypt [**Fig 3D**].

### High CHI3L1 expression correlates with an increase in colonic immune cell infiltration at steady state in MOLF mice

**Fig 4:**
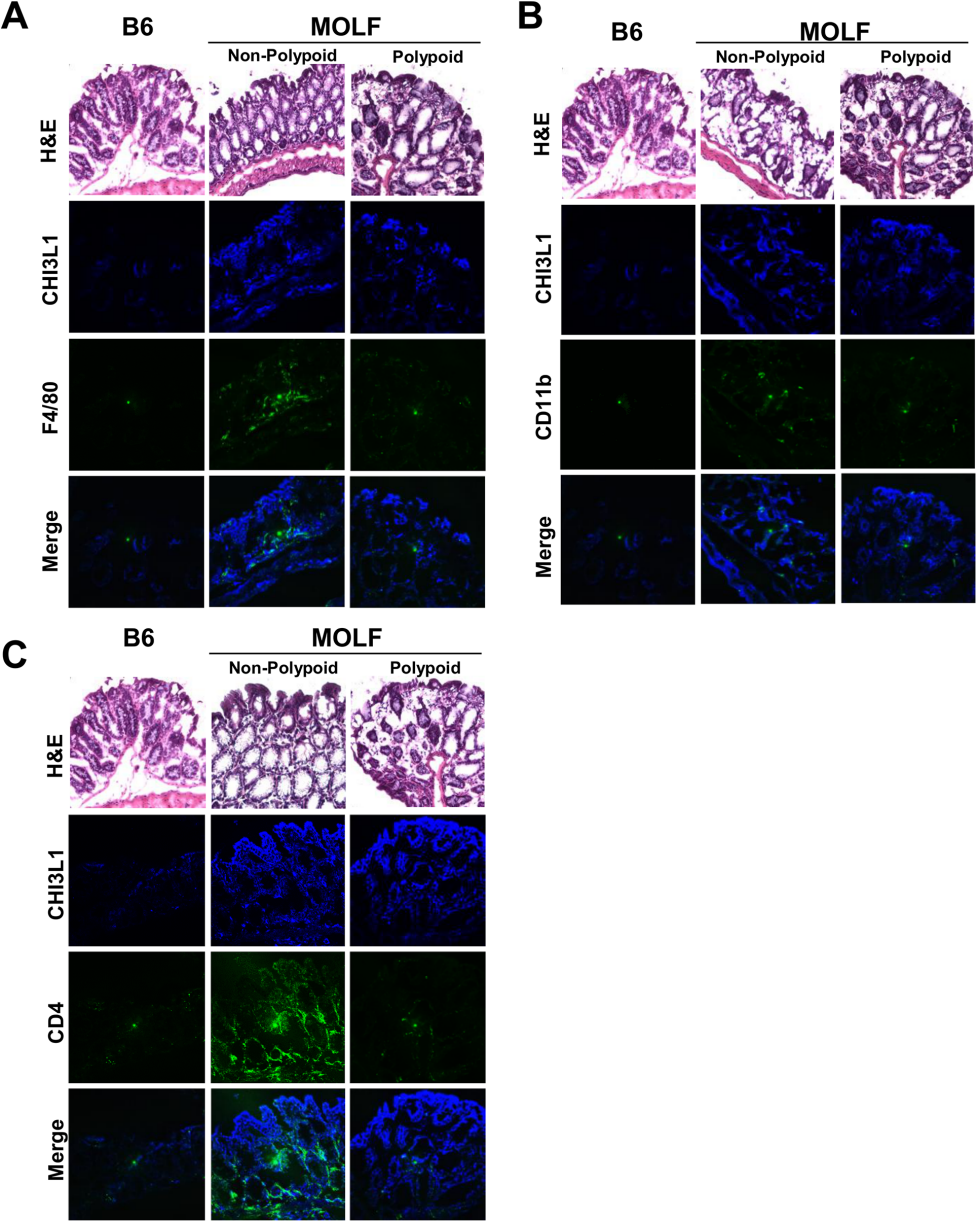
MOLF express high levels of CHI3L1 in the colon and polypoid area and associates with high immune cellular infiltrates. Two-color Immunofluorescence analysis was performed on colonic sections from six months old B6 and MOLF mice using anti-CHI3L1 and anti-F4/80 (**A**) -CD11b (**B**) or -CD4 (**C**) primary antibodies respectively.

To visualize the extent of high CHI3L1 expression in the MOLF, colonic sections were subjected to immunofluorescence staining using anti-CHI3L1 primary and Alexa–647 secondary antibodies. Notably, CHI3L1 was barely detectable in the colon of B6 mice [**Fig 4A, 4B and 4C**]. In contrast, MOLF mice showed high CHI3L1 expression throughout the mucosal and sub-mucosal areas. In the polypoid nodules of the MOLF mice, CHI3L1 expression was predominately localized at the tip of the epithelial layer, which highly co-localized with the Ki–67 positive cells [**Fig 3D**].

**Fig 5:**
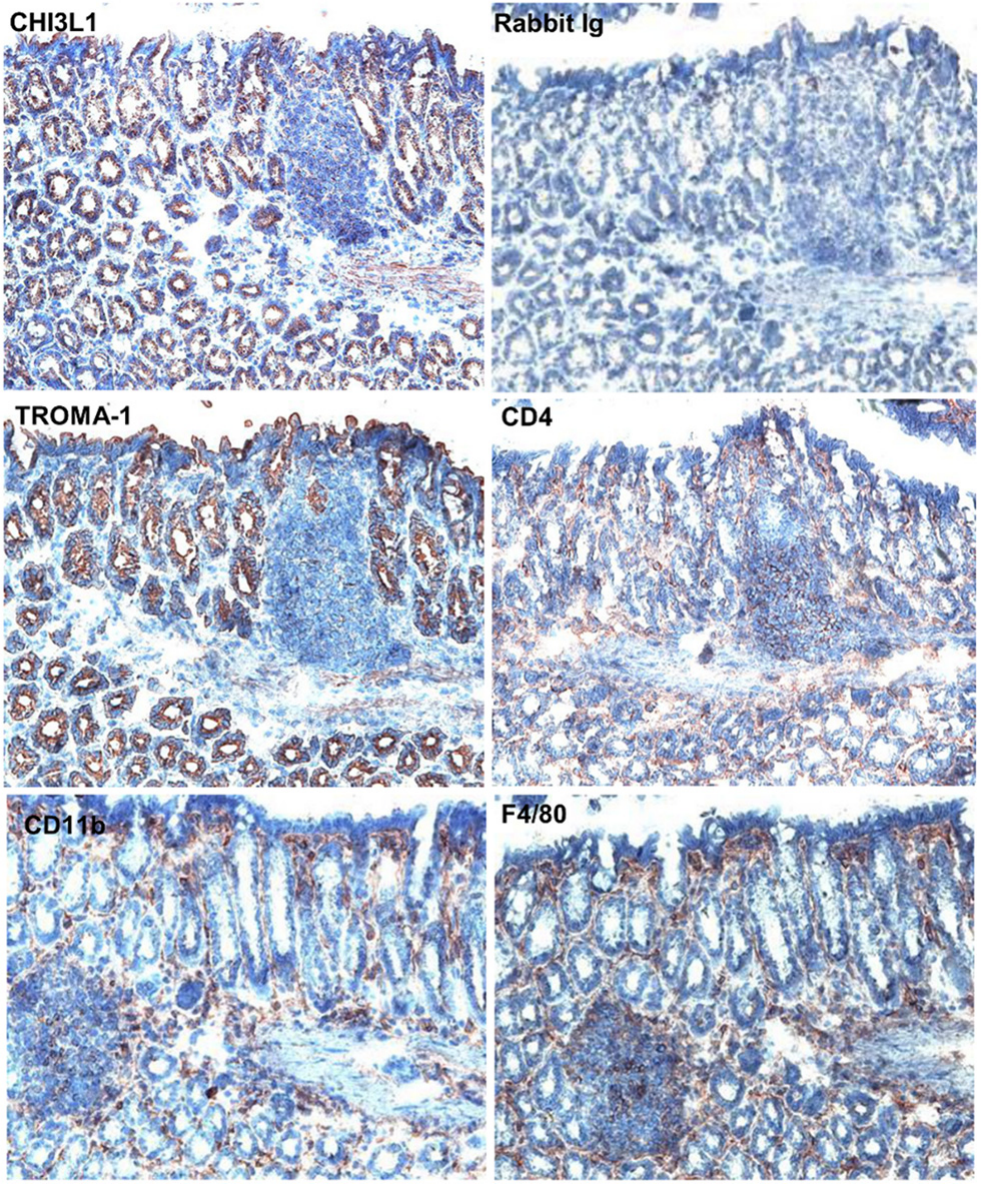
MOLF express high levels of CHI3L1 in colonic epithelial cells but not in immune cells. Immunohistochemical analysis was performed on colonic serial sections (non-polypoid area) from six months old MOLF mice using anti-CHI3L1, -TROMA–1 (cytokeratin 8), -CD4, -CD11b, and -F4/80 antibodies.

Since CHI3L1 has been previously shown to directly enhance immune cell recruitment *in vitro*, both MOLF and B6 mice derived colonic sections were co-stained with anti-F4/80, -CD11b, -CD4 primary and FITC-conjugated secondary antibodies. The serial sections were stained with H&E to show the morphology of each colonic section. The high CHI3L1 expression in the MOLF mice was associated with significantly increased numbers of immune cell infiltrations in the colon at the steady state [**Fig 4A, 4B and 4C**]. In contrast, B6 mice did not show appreciable infiltration of these immune cells in the colon [**Fig 4A, 4B and 4C**]. As shown in **Fig 5**, CHI3L1 was mainly expressed in TROMA–1 (cytokeratin–8) positive epithelial cells but not in F4/80^+^ macrophages, CD11b^+^ granulocytes/macrophages/myeloid dendritic cells or CD4^+^ T cells in MOLF-derived colonic sections. Furthermore, two-color immunohistochemistry using the serial sections has confirmed that CHI3L1 is specifically expressed on the TROMA–1 positive CECs [**Fig A and Fig B in S1 Fig**].

### AOM-induced colorectal cancer was significantly more frequently developed in MOLF mice than B6 mice

**Fig 6:**
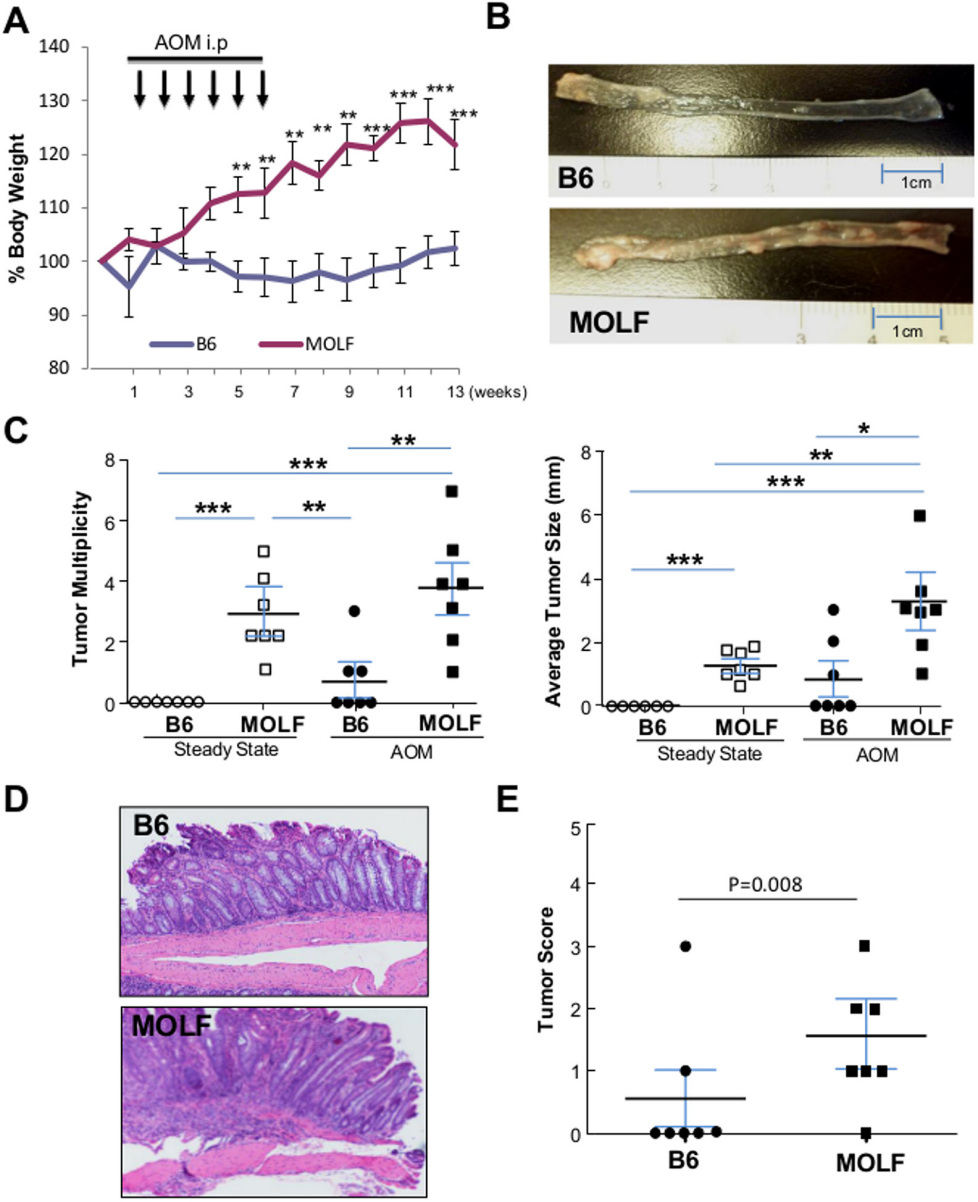
MOLF mice showed exacerbated AOM-induced CRC development. [**A**] AOM was intraperitonally injected into 6 months old B6 (n = 7) and MOLF (n = 7) mice once a week for 6 weeks and sacrificed after addition 8 weeks. Body weight was measure weekly as shown. [**B**] AOM-treated B6 and MOLF mice colons were open-up longitudinally and inspected. [**C**] Tumor size and multiplicity of colons from steady state or AOM-treated B6 and MOLF mice were determined under a dissection microscope (n = 7 in each group). [**D**] Representative H&E section of colons from AOM-treated B6 and MOLF mice are shown. [**E**] Histological scoring of tumor from AOM-treated B6 (n = 7) and MOLF (n = 7) mice are shown. *P<0.05, **P<0.01, ***P<0.001 between the two groups as indicated.

MOLF mice developed spontaneous polypoid nodules at the steady state, but since these nodules did not display the characteristics of dysplastic transformation at steady conditions, we next examined the susceptibility of MOLF mice to carcinogenesis under chronic inflammatory conditions. To do so, 8–10 weeks old MOLF and B6 mice were given intraperitoneal injections of AOM (12mg/Kg) once a week for 6 weeks, and were sacrificed 8 weeks after the last injection. During the treatment, B6 mice did not show significant body weight fluctuations, but MOLF mice continuously increased body weight [**Fig 6A**]. However when the colons were dissected longitudinally at the end of experiment, MOLF mice showed striking development of polypoid nodules as compared to B6 mice [**Fig 6B**]. Both polypoid nodule number and size were significantly larger in the MOLF mice than in the B6 mice [**Fig 6C**]. In addition, histological scoring of the H&E sections revealed significantly higher grade of tumors in the MOLF mice, suggesting that CHI3L1 plays a role in tumorigenesis by promoting larger number and size of more advanced tumors [**Fig 6D and 6E**].

## Discussion

The genetic diversity present in wild-derived mice increases the likelihood of phenotypic variations as compared to classical mice strains. This allows experimental interrogation to explore novel phenotypes. Traditionally, wild-derived mice tend to be underrepresented in forward genetic studies. However, several disease-related genes have been discovered using wild-derived mouse strains, such as the Mx1 and MnV genes that confer resistance of wild-derived mice to influenza virus and play a role in West Nile virus infection pathogenesis respectively [**13, 14**]. More recently, hypo-responsiveness to polyinosinic-polycytidylic acid [poly(I:C)] stimulation was found in a wild-derived MSM/Ms mouse strain that exhibits mutations in the toll-like receptor 3 (TLR3) [**15**]. Likewise, peritoneal macrophages in MOLF mice show hypo-responsiveness to TLR9-ligand, CPG-motif containing oligodeoxynucleotides (CpG ODNs) although these mice are fully responsive to other endosomal TLR agonists [**16**]. The hypo-responsiveness to CpG ODNs in MOLF mice is mainly a consequence of the specific mutations in the mannose receptor 1 (mrc1) gene, resulting in low expression levels of MRC1, which is dispensable for CpG-induced cytokine responses [**16**]. Since wild-derived MOLF mice show a divergent development of genes, which are activated in response to pathogens and their microbial components, this mouse model seems to be one of the most useful tools for further understanding natural occurring regulators in innate immune responses under inflammatory conditions [**16, 17**].

Although the main advantage of wild-derived mice is the heterogeneity within the population that allows linkage analysis to identify disease-causing genes, these mice have to be wildly caught and examined in order to leverage on this advantage. However, inbreeding these wild-derived mice line results in the loss of the heterogeneity [**18**]. Despite this, the “genetic deviants” present in these strains, when compared to common laboratory strains, allow one to (re)discover phenotype-causing genes. In the case of this study, the polymorphisms in the CHI3L1 proximal promoter render higher transcriptional activation in the MOLF mice at steady state. This is in contrast to the colonic expression of CHI3L1 in B6 mice, which is only induced during inflammatory and tumorigenenic conditions. The high expression of this proliferation-related gene in the MOLF mice colon may associate with the spontaneous development of protrusive polypoid nodules.

Some previous reports have demonstrated that both promoter and intragenic polymorphisms in human CHI3L1 is associated with serum CHI3L1 levels and disease severity, such as bronchial asthma [**19**]. However, multiple alignments between the CHI3L1 intronic SNPs of B6 and MOLF mice do not show any distinct pattern conservation with human counterparts [**S2 and S3 Figs**]. This may be a result of lesser conservation of introns in general, as compared to gene promoters and exons. Therefore, in this study, we have focused on the effects of the promoter SNPs in MOLF mice, demonstrating higher CHI3L1 levels and conservation with the human counterpart. Bhardwaj et al has previously reported that IL–1 and IL–6 family cytokines-driven expression of CHI3L1 in astrocytes required both STAT3 and NF-κB binding elements of the CHI3L1 promoter [**20**]. However, the binding sites of these two transcription factor are outside the region of the SNPs analyzed in this study (-1 to -579 bp). Since our group demonstrated that the SNPs within this region (-1 to -517 bp) showed an effect on CHI3L1 expression, it is therefore unlikely to be a result of transcription factor binding. Rather than that, we have noticed that many of the promoter-SNPs, between B6 and MOLF mice, are occurring at the region of CG dinucleotides. This may potentially affect proper DNA methylation, and the higher CHI3L1 gene activation in MOLF mice. In our future study, more detailed characterization of CHI3L1 intronic regions will be carried out to identify potential enhancer sites, splicing changes, or presence of small regulatory RNAs, to determine if these murine intronic SNPs are functional.

Despite CECs showing hyperproliferation in the MOLF mice that may be responsible for the protrusions, these CECs do not exhibit cytological abnormalities or neoplastic changes at steady state. This finding may be consistent with our unpublished observations that CHI3L1 does not directly participate in neoplastic transformation in the colon, but rather promotes CECs survival and proliferation during intestinal inflammation and tumorigenesis in B6 mice. Therefore, once tumor formation was induced by carcinogen (AOM) injection, inherently increased CHI3L1 expression in MOLF mice allows drastically accelerated tumor growth than B6 mice with increased polypoid nodule numbers and sizes.

In addition to the aberrant CEC proliferation in MOLF mice, previous studies have reported other characteristics in these mice that are associated with the major CHI3L1 protein functions. For instance, MOLF mice are extremely susceptible to Salmonella infection, as compared to other inbred strains such as A/J or B6 x MOLF F1 strains [**21, 22**]. Our group has also reported that CHI3L1 can directly increase mice susceptibility to Salmonella, and other potentially pathogenic *E coli*, by enhancing its adhesion and invasion on/into CECs [**2,12**]. Therefore, it is possible that enhanced bacterial/epithelial interaction due to increased CHI3L1 expression makes MOLF mice more susceptible to carcinogenesis and tumor cell expansion. Indeed, it has been reported that enteric microbiota can release factors that further promote tumor cell expansion [**23**].

Taken together, the wild-derived MOLF mouse strain is an ideal model to understand the mechanism of tumor expansion and proliferation in CRC. High endogenous CHI3L1 expression in the steady state of MOLF mice associates with high proliferation rates in the colon and may promote spontaneous development of polypoid protrusion in the colon. This results in the rapid expansion of tumor cells when CRC is induced in the mice.

## Supporting Information

S1 Fig Two-color immunohistochemical analysis with anti-CHI3L1 and anti-cytokeratin 8 antibodies in MOLF colon. (TIFF)

S2 Fig Alignment of Human, MOLF/EiJ (MOLF) and C57Bl/6 (B6) mice CHI3L1 SNPs in intron 5. (TIFF)

S3 Fig Alignment of Human, MOLF/EiJ (MOLF) and C57Bl/6 (B6) mice CHI3L1 SNPs in intron 6. (TIFF)

S1 Text Supporting Materials & Methods. (DOC)

S2 Text Supporting Figure Legends. (DOC)

S3 Text Supporting References. (DOC)
