# Supplementary figures and images for: High Endogenous Expression of Chitinase 3-Like 1 and Excessive Epithelial Proliferation with Colonic Tumor Formation in MOLF/EiJ Mice
Source: PLoS One. 2015 Oct 6;10(10):e0139149. doi: 10.1371/journal.pone.0139149 (PMC4594921; doi:10.1371/journal.pone.0139149)

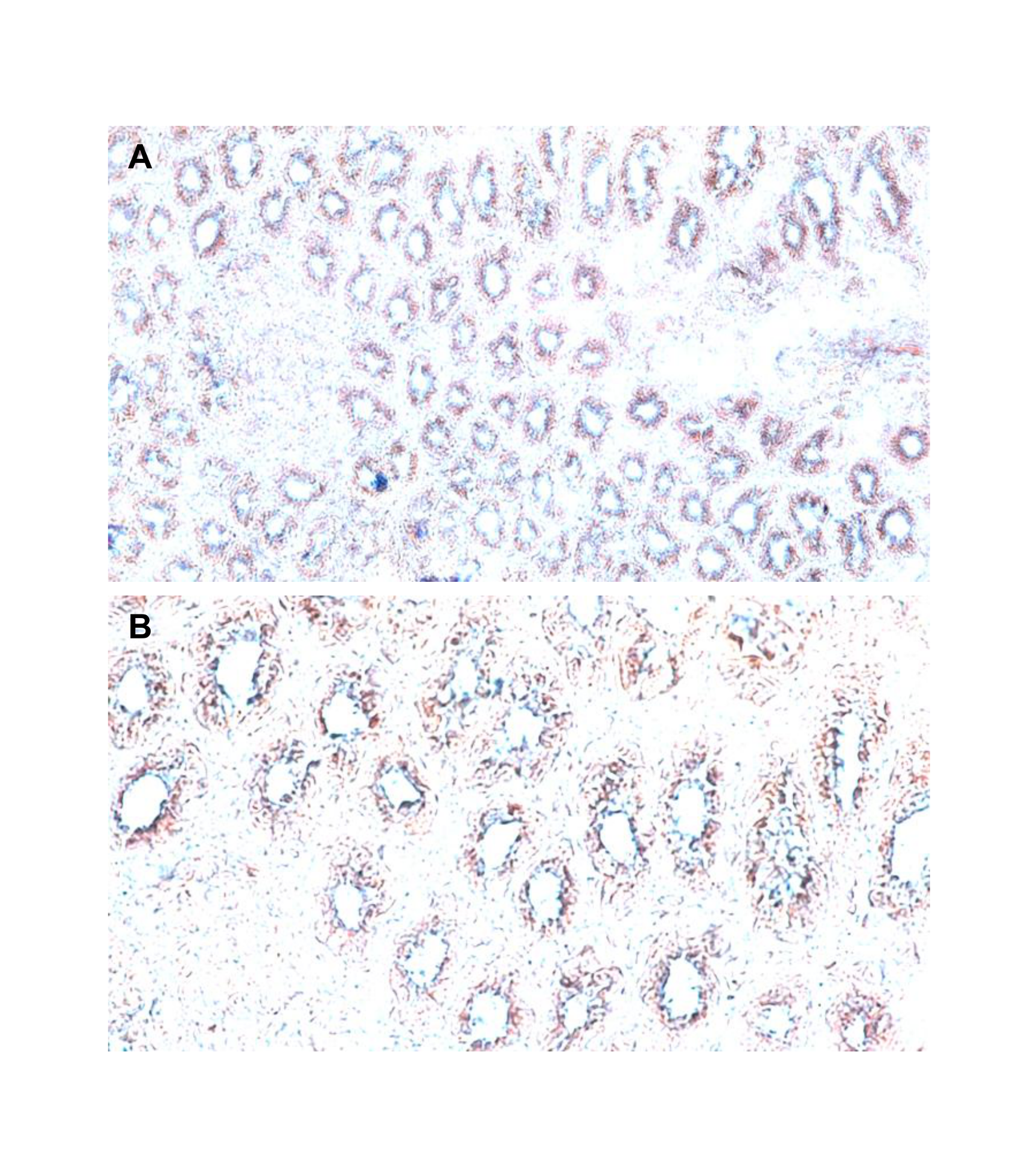

Supplement: S1 Fig — (TIFF) [file pone.0139149.s001.tiff]

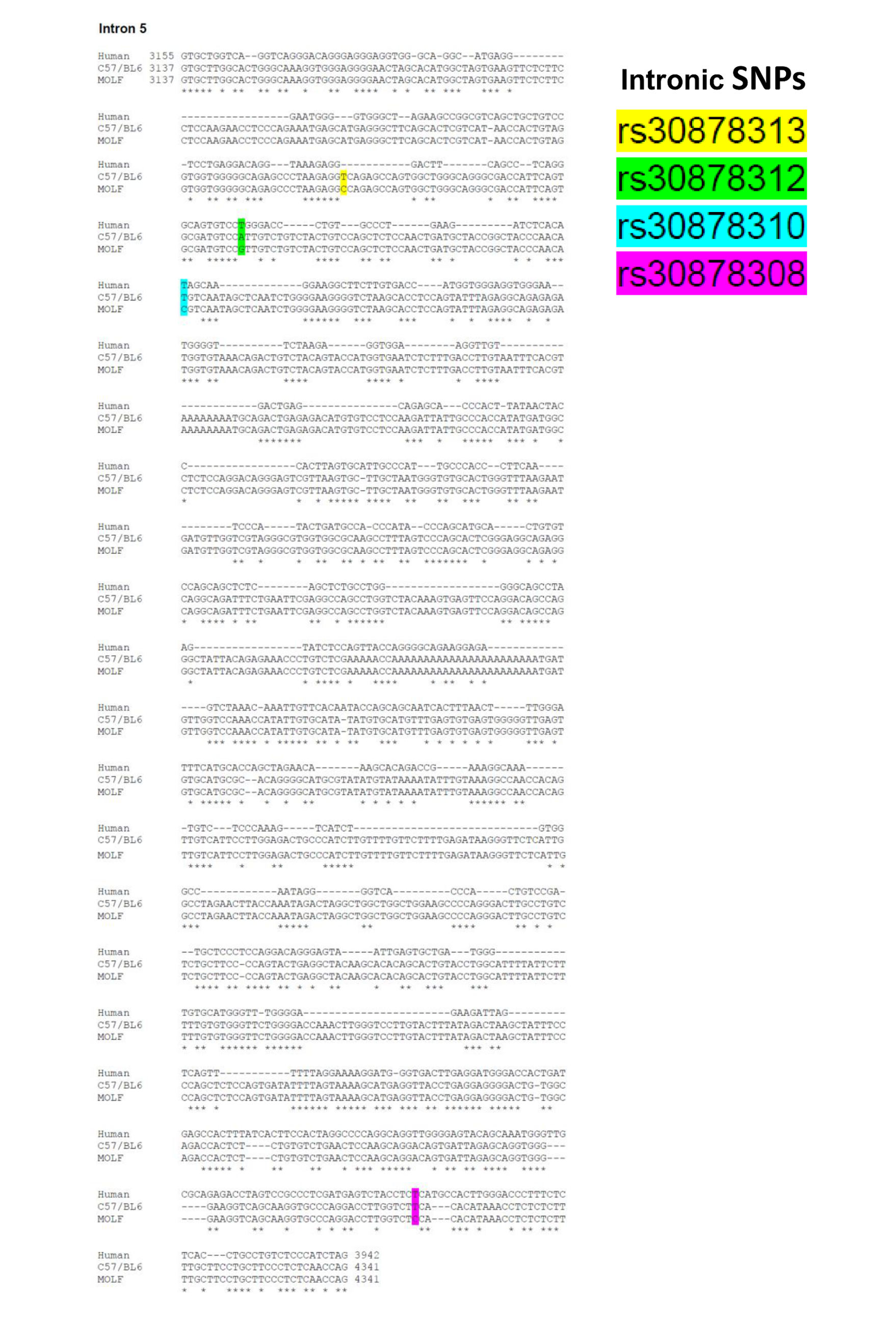

Supplement: S2 Fig — (TIFF) [file pone.0139149.s002.tiff]

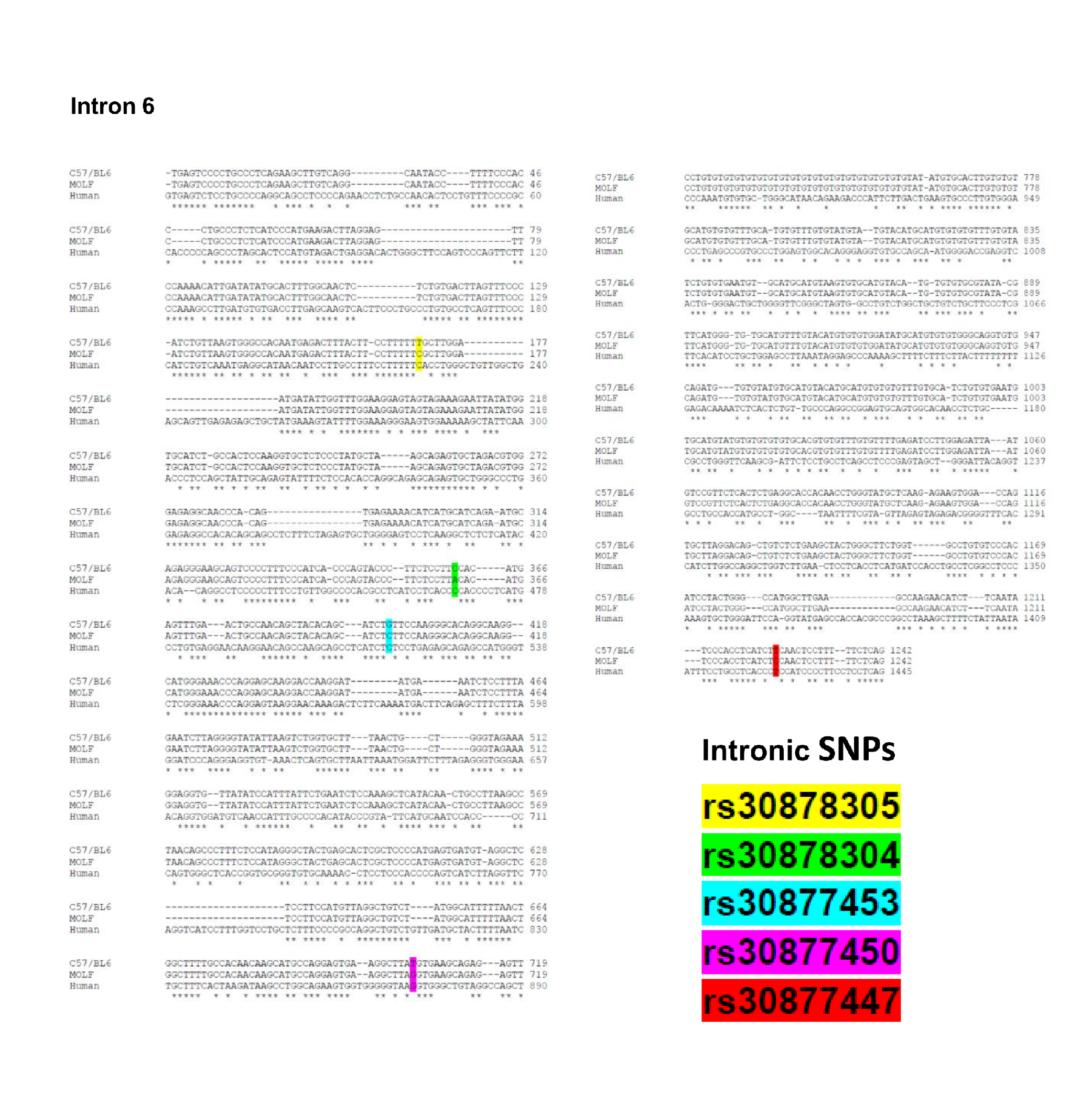

Supplement: S3 Fig — (TIFF) [file pone.0139149.s003.tiff]
