# Supplementary material for: High Endogenous Expression of Chitinase 3-Like 1 and Excessive Epithelial Proliferation with Colonic Tumor Formation in MOLF/EiJ Mice
Source: PLoS One. 2015 Oct 6;10(10):e0139149. doi: 10.1371/journal.pone.0139149 (PMC4594921; doi:10.1371/journal.pone.0139149)
Supplement: S1 Text — (DOC) [file pone.0139149.s004.doc]

**Supporting Materials and Methods**

**Two-color immunohistochemical analysis**

Two-color immunohistochemical analysis was performed as previously described with the following modification [**Figure A in S3 Text**]. 4 μm thick specimens were fixed in acetone for 10 minutes, air-dried, and incubate with anti-CHI3L1 antibody (Affinity Bioreagent, Golden, CO) for 1 hour at room temperature. For detection, biotinylated goat anti-rabbit IgG (Vector Laboratories, Inc., Burlingame, CA) was used, followed by avidin-biotinylated peroxidase complex (Dako Corp., Santa Barbara, CA). Each step was followed by three washes with PBS. The specimens were developed in a solution of 3-amino-9 ethylcarbazole (AEC: Aldrich Chemical Co., Milwaukee, WI); the reaction was stopped by dipping the specimen in distilled water for 5 minutes and washing it in PBS for 10 min. The specimens were then incubated with anti-Cytokeratine 8, TROMA-1 antibody (Developmental Studies Hybridoma Bank at the University of Iowa (Iowa City, IA) at room temperature for 1 hour. For detection, biotinylated rabbit anti-rat IgG was used, followed by incubation with avidin-biotinylated alkaline-phosphatase complex (Vector). After development with blue substrate kit (Vector), the specimens were washed with water, postfixed with 2% paraformaldehyde and mounted with Glycergel (Dako). Incubation with 0.3% H_2_O_2_ in PBS was used to block endogenous peroxidase activity.
