# Supplementary material for: High Endogenous Expression of Chitinase 3-Like 1 and Excessive Epithelial Proliferation with Colonic Tumor Formation in MOLF/EiJ Mice
Source: PLoS One. 2015 Oct 6;10(10):e0139149. doi: 10.1371/journal.pone.0139149 (PMC4594921; doi:10.1371/journal.pone.0139149)
Supplement: S2 Text — (DOC) [file pone.0139149.s005.doc]

**Supporting Figure Legend**

**S1 Fig: Two-color immunohistochemical analysis with anti-CHI3L1 and anti-cytokeratin 8 antibodies in MOLF colon.**

Two-color immunohistochemistry shows CHI3L1-positive cells (red), cytokeratin 8-positive cells (blue) and CHI3L1^+^/cytokeratin 8^+^ cells (purple) in colon from 6 months old MOLF mice with steady state. CHI3L1 is specifically expressed on the TROMA-1 positive epithelial cells. Objective, 20x [**A**] and 40x [**B**].

**S2 Fig: Alignment of Human, MOLF/EiJ (MOLF) and C57Bl/6 (B6) mice CHI3L1 SNPs in intron 5**

Multiple alignments between the CHI3L1 SNPs in the intron 5 of B6 and MOLF mice do not show any distinct pattern conservation with human counterparts.

**S3 Fig: Alignment of Human, MOLF/EiJ (MOLF) and C57Bl/6 (B6) mice CHI3L1 SNPs in intron 6**

Multiple alignments between the CHI3L1 SNPs in the intron 6 of B6 and MOLF mice do not show any distinct pattern conservation with human counterparts.
