# Supplementary material for: High Endogenous Expression of Chitinase 3-Like 1 and Excessive Epithelial Proliferation with Colonic Tumor Formation in MOLF/EiJ Mice
Source: PLoS One. 2015 Oct 6;10(10):e0139149. doi: 10.1371/journal.pone.0139149 (PMC4594921; doi:10.1371/journal.pone.0139149)
Supplement: S3 Text — (DOC) [file pone.0139149.s006.doc]

**Supporting References**

1. Mizoguchi A, Mizoguchi E, Chiba C, Spiekermann GM, Tonegawa S, et al. (1996) Cytokine inbalance and autoantibodies production in T cell receptor-α mutant mice with inflammatory bowel disease. J Exp Med 183: 847-856.
